# Mechanism of the Crepitant and Subcrepitant Rale

**Published:** 1869-11

**Authors:** 


### Miscellaneous.

### Mechanism of the Crepitant and Subcrepitant Rale.

At a meeting of the New York Medical Journal Association, held December 18th, Dr. Austin Flint read a paper on *The Mechanism of the Crepitant and the Subcrepitant Rale*, (*N. Y. Medical Journal*, Feb., 1869, vol. viii., p. 449). He showed that these rales could be perfectly imitated by means of the article known as "patent indiarubber sponge" lately introduced. Compressing the sponge and letting it expand near the ear—dry for the crepitant, and moist for the subcrepitant, rale—the latter would be heard during both compression and expansion, the former during expansion only. This illustrated the true theory of the crepitant rale, that it is caused by the sudden separation of coherent surfaces, and not by bubbling—an explanation first propounded by Dr. Edson Carr, of Canandaigua, N.Y., in 1842, (*Am. Jour. Med. Sci.*, Oct., 1842, p. 360.) The history of opinion upon this subject, from the time of Lænnec to the present, was reviewed; showing that most authorities still adhered to the "bubbling" theory, though a few had adopted Carr's explanation without giving him credit for priority. The important points of this valuable paper were summarized in conclusion, as follows:

"1. The crepitant rale is caused by the separation of the walls of the air-vesicles and bronchioles, in the manner explained by the late Dr. Edson Carr.

"2. It is highly probable that the peculiar ('vesicular') quality pertaining to the inspiratory sound in the healthy murmer of respiration is due to the same cause, the cohesion of the walls of the air-vesicles and bronchioles not being sufficient to give rise to the crepitant rale.

"3. The subcrepitant rale is caused by the bubbling of liquid in the minute bronchial tubes, and also in the air-vesicles and bronchioles.

"4. The essential distinctive character of the crepitant rale is its dryness. The term 'dry crackling' expresses this character, whereas the phrase 'fine bubbling' expresses the character of the sound in the sub-crepitant rale. In addition, the crepitant rale is not produced in expiration as well as in inspiration.

"5. Very fine bubbling due to liquid in air-vesicles and bronchioles resembles the fine crackling sound which characterizes the crepitant rale; and the discrimination of the former from the latter requires a nice perception of differences in sound, and some practice in comparing the two rales The artificial production of the two rales (by means of the 'rubber-sponge') may be made highly useful by affording this practice.

"6. The crepitant and the subcrepitant rale are not infrequently found in combination. They are likely to be conjoined whenever the air-vesicles and bronchioles contain liquid of any kind.

"7. In view of the fact that for the artificial production of the crepitant rale no liquid is necessary, and in view of the fact that for the production of fine bubbling sounds an extremely small amount of liquid only is required, wherever in disease a crepitant rale exists, without the co-existence of the subcrepitant rale, it is probable that there is a morbid adhesiveness of the inner surface of the air-vesicles and bronchioles, without the presence of an appreciable amount of liquid. Hence, in the cases in which the crepitant rale exists alone, either in the first stage of pneumonia or in the resolving stage, the morbid product within the air vesicles and bronchioles must be either a glutinous matter sufficient to give adhesiveness to the wall, but not enough for bubbling, or the product is a semi-solid, in which bubbles are not readily produced; and in cases of œdema ot the lungs, or when blood is present in the air-vesicles and bronchioles, the crepitant rale can hardly be expected to occur without being associated with the subcrepitant.

"8. The characters of the subcrepitant rale are materially the same, although the bubbling is produced in liquids differing as regards consistence."—*Medical Record.*

